# Phylogeography of Lassa Virus in Nigeria

**DOI:** 10.1128/JVI.00929-19

**Published:** 2019-10-15

**Authors:** Deborah U. Ehichioya, Simon Dellicour, Meike Pahlmann, Toni Rieger, Lisa Oestereich, Beate Becker-Ziaja, Daniel Cadar, Yemisi Ighodalo, Thomas Olokor, Emmanuel Omomoh, Jennifer Oyakhilome, Racheal Omiunu, Jacqueline Agbukor, Benevolence Ebo, John Aiyepada, Paulson Ebhodaghe, Blessing Osiemi, Solomon Ehikhametalor, Patience Akhilomen, Michael Airende, Rita Esumeh, Ekene Muoebonam, Rosemary Giwa, Anieno Ekanem, Ganiyu Igenegbale, George Odigie, Grace Okonofua, Racheal Enigbe, Edna Omonegho Yerumoh, Elisa Pallasch, Sabrina Bockholt, Liana E. Kafetzopoulou, Sophie Duraffour, Peter O. Okokhere, George O. Akpede, Sylvanus A. Okogbenin, Ikponmwosa Odia, Chris Aire, Nosa Akpede, Ekaete Tobin, Ephraim Ogbaini-Emovon, Philippe Lemey, Donatus I. Adomeh, Danny A. Asogun, Stephan Günther

**Affiliations:** a Bernhard Nocht Institute for Tropical Medicine, Hamburg, Germany; b Department of Biological Sciences, Redeemer’s University, Ede, Nigeria; c Department of Microbiology, Faculty of Life Sciences, Ambrose Alli University, Ekpoma, Nigeria; d Irrua Specialist Teaching Hospital, Irrua, Nigeria; e Department of Microbiology, Immunology and Transplantation, Rega Institute, KU Leuven, Leuven, Belgium; f Spatial Epidemiology Lab (SpELL), Université Libre de Bruxelles, Brussels, Belgium; g German Center for Infection Research (DZIF), Partner site Hamburg–Lübeck–Borstel–Riems, Hamburg, Germany; h National Infections Service, Public Health England, Porton Down, United Kingdom; i NIHR Health Protection Research Unit in Emerging and Zoonotic Infections, University of Liverpool, Liverpool, United Kingdom; University of Texas Southwestern Medical Center

**Keywords:** Lassa virus, Nigeria, phylogeny

## Abstract

Lassa virus is genetically diverse with several lineages circulating in West Africa. This study aimed at describing the sequence variability of Lassa virus across Nigeria and inferring its spatiotemporal evolution. We sequenced and isolated 77 Lassa virus strains from 16 Nigerian states. The final data set, including previous works, comprised metadata and sequences of 219 unique strains sampled between 1969 and 2018 in 22 states. Most of this data originated from Lassa fever patients diagnosed at Irrua Specialist Teaching Hospital, Edo State, Nigeria. The majority of sequences clustered with the main Nigerian lineages II and III, while a few sequences formed a new cluster related to Lassa virus strains from Hylomyscus pamfi. Within lineages II and III, seven and five sublineages, respectively, were distinguishable. Phylogeographic analysis suggests an origin of lineage II in the southeastern part of the country around Ebonyi State and a main vector of dispersal toward the west across the Niger River, through Anambra, Kogi, Delta, and Edo into Ondo State. The frontline of virus dispersal appears to be in Ondo. Minor vectors are directed northeast toward Taraba and Adamawa and south toward Imo and Rivers. Lineage III might have spread from northern Plateau State into Kaduna, Nasarawa, Federal Capital Territory, and Bauchi. One sublineage moved south and crossed the Benue River into Benue State. This study provides a geographic mapping of lineages and phylogenetic clusters in Nigeria at a higher resolution. In addition, we estimated the direction and time frame of virus dispersal in the country.

**IMPORTANCE** Lassa virus is the causative agent of Lassa fever, a viral hemorrhagic fever with a case fatality rate of approximately 30% in Africa. Previous studies disclosed a geographical pattern in the distribution of Lassa virus strains and a westward movement of the virus across West Africa during evolution. Our study provides a deeper understanding of the geography of genetic lineages and sublineages of the virus in Nigeria. In addition, we modeled how the virus spread in the country. This knowledge allows us to predict into which geographical areas the virus might spread in the future and prioritize areas for Lassa fever surveillance. Our study not only aimed to generate Lassa virus sequences from across Nigeria but also to isolate and conserve the respective viruses for future research. Both isolates and sequences are important for the development and evaluation of medical countermeasures to treat and prevent Lassa fever, such as diagnostics, therapeutics, and vaccines.

## Abstract (precis)

Lassa virus is the causative agent of Lassa fever, a viral hemorrhagic fever with a case fatality rate of approximately 30% in Africa. Previous studies disclosed a geographical pattern in the distribution of Lassa virus strains and a westward movement of the virus across West Africa during evolution. Our study provides a deeper understanding of the geography of genetic lineages and sublineages of the virus in Nigeria. In addition, we modeled how the virus spread in the country. This knowledge allows us to predict into which geographical areas the virus might spread in the future and prioritize areas for Lassa fever surveillance. Our study not only aimed to generate Lassa virus sequences from across Nigeria but also to isolate and conserve the respective viruses for future research. Both isolates and sequences are important for the development and evaluation of medical countermeasures to treat and prevent Lassa fever, such as diagnostics, therapeutics, and vaccines.

## INTRODUCTION

Lassa fever is a febrile illness in West Africa. Severe infections are associated with bleeding, central nervous system manifestations, and renal failure (1). The case fatality rate in the current hospital setting in Africa is around 30% (2, 3). The causative agent—Lassa virus—is endemic in the West African countries of Guinea, Sierra Leone, Liberia, Mali, Côte d’Ivoire, and Nigeria (4–6). Individual cases of Lassa fever have also been observed in Benin and Togo, though endemicity of the virus in these countries needs to be confirmed (7).

Lassa virus is genetically diverse, and several lineages may be distinguished. Four lineages have been initially described by Bowen et al. (4) as follows: lineages I, II, and III circulating in Nigeria and lineage IV circulating in Guinea, Sierra Leone, and Liberia. Strains from Mali and Côte d’Ivoire were proposed to represent lineage V (8). The main natural reservoir appears to be Mastomys natalensis, although Mastomys erythroleucus has recently been described as an alternative host for lineages III and IV (9–13). The newly discovered Lassa virus strain Kako from a Hylomyscus pamfi rodent that was trapped in Nigeria and the virus from the nosocomial outbreak in Togo may be considered lineages VI and VII, respectively (7, 12). Preliminary evidence for a putative further lineage has been provided by two short virus sequences obtained from patients in the south of Nigeria (14).

Since 2008, a laboratory for the molecular diagnostics of Lassa fever has been in operation at the Irrua Specialist Teaching Hospital (ISTH), Irrua, in Edo State, Nigeria (2). As it is one of a few centers in West Africa providing this service, it has become a reference center for Lassa fever in the country. About 1,000 to 4,000 suspected cases are tested annually with an average confirmation rate of about 10%. While the vast majority of confirmed Lassa fever cases originated from Edo State, a significant number of specimens originated from patients attending hospitals in other parts of the country.

Previous studies have provided a comprehensive overview of the sequence variability of the viruses circulating in the catchment area of ISTH (11). Complete sequence information for Lassa virus strains from other parts of the country is scarce (4, 14). Therefore, this study aimed to describe the sequence variability of Lassa virus across Nigeria and infer the temporal and spatial evolution of the virus in the country. Virus strains from patients who were diagnosed with Lassa fever at ISTH and presumably acquired the infection outside of Edo State were sequenced using next-generation sequencing (NGS) technology, and the sequences were subjected to phylogeographic analysis.

## RESULTS

Samples from PCR-confirmed Lassa fever patients who had attended hospitals in 16 states of Nigeria were selected for virus isolation and sequencing. Seventy-two Lassa virus strains were isolated in cell culture. Complete S and L segment coding sequences were obtained for 72 and 55 isolates, respectively. If virus isolation was not successful, the virus was sequenced directly from clinical material generating another 2 and 12 complete and partial S segment coding sequences, respectively. In the phylogeographic reconstruction, we included partial or complete sequences from this study (NCBI BioProject accession number PRJNA482054), our 2018 sequencing project at ISTH (NCBI BioProject accession number PRJNA482058) (15), and GenBank, for which the residence of the patient or hospital where the patient had been treated or the trapping site in cases of animals has been on record at least at the level of the State in Nigeria. The final data set comprised sequences and metadata for 219 unique Lassa virus strains sampled between 1969 and 2018 in 22 Nigerian states (216 sequences for the S segment and 157 sequences for the L segment) (see Table S1 in the supplemental material).

**FIG 1:**
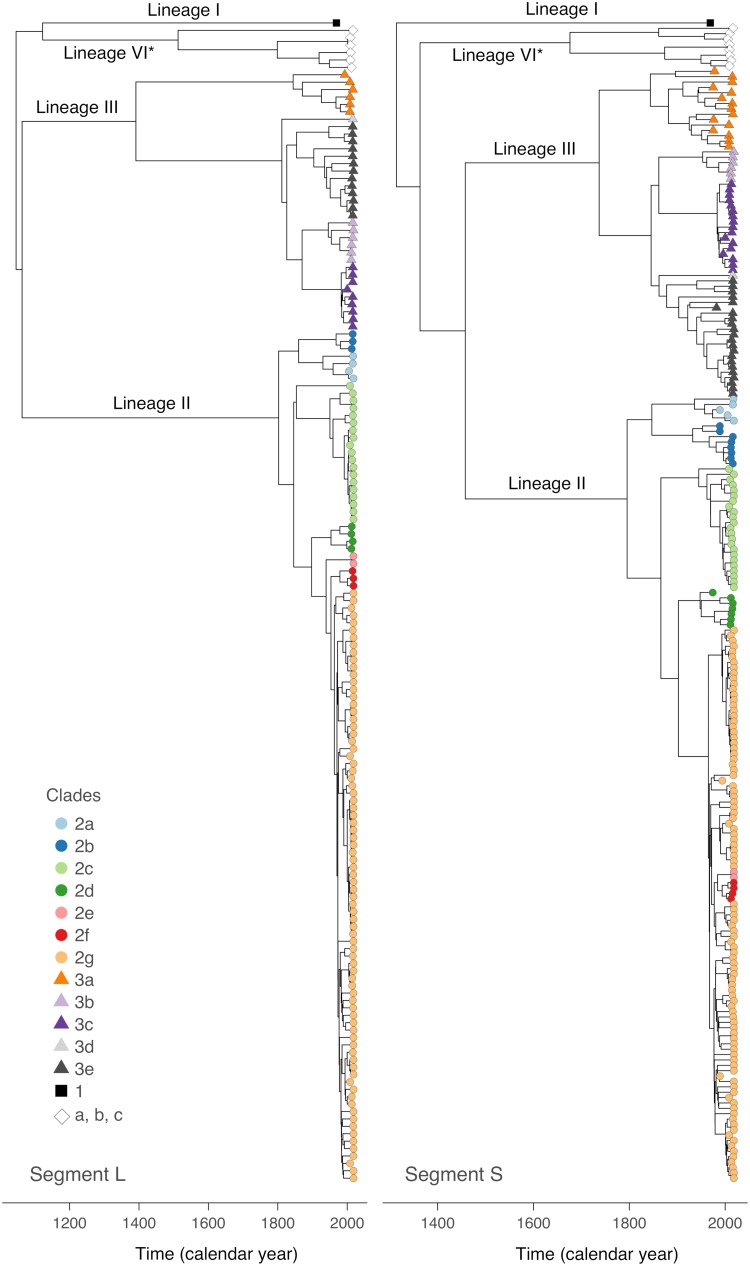
Time-scaled maximum clade credibility trees for S and L segments. The evolutionary relationships among the Lassa virus lineages in Nigeria were investigated by BEAST analysis using a simple constant population size model. Detailed phylogenies of lineages II and III based on a flexible skygrid coalescent model are shown in Fig. S3 to S6 in the supplemental material. The sublineages (clades 2a through 2g and 3a through 3e for lineages II and III, respectively) have been defined according to the phylogenies inferred separately for lineages II and III and geographic location of the strains (see Fig. S3 to S8 in the supplemental material). Detailed phylogenies of lineage I (clade 1) and the cluster including Lassa virus strains from *H. pamfi* (lineage VI, clade c) and related human sequences (clades a and b) are shown in Fig. S1 and S2 in the supplemental material. The molecular clock rate estimated for the L and S tree was 7.8 × 10^−4^ substitutions/(site × year) (95% highest posterior density interval [5.4, 10.2]) and 7.8 × 10^−4^ substitutions/(site × year) (95% highest posterior density interval [6.3, 9.1]), respectively. *, tentative lineage designation.

**FIG 2:**
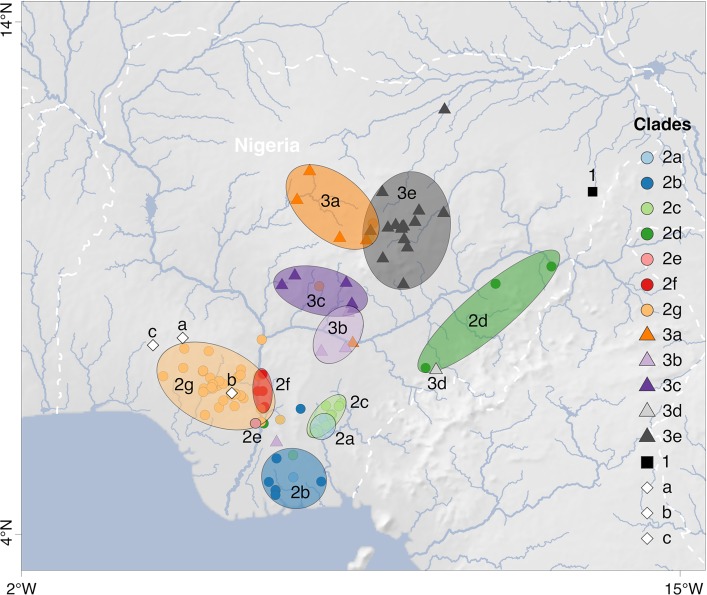
Map of Lassa virus lineages and sublineages in Nigeria. The putative core areas for circulation of sublineages, i.e., areas with the highest density of strains belonging to a certain sublineage, are encircled and colored according to the clade. Clades 2a through 2g and 3a through 3e represent the sublineages of lineages II and III, respectively (see Fig. S3 to S6 in the supplemental material). Clade 1 and clades a, b, and c represent lineage I and the cluster including Lassa virus strains from *H. pamfi* and related human sequences, respectively (see Fig. S1 and S2 in the supplemental material). Each symbol on the map marks a single strain or several strains with the same coordinates. Separate maps for each sublineage within lineages II and III are shown in Fig. S7 and S8 in the supplemental material; for coordinates of each strain see Table S1 in the supplemental material. Map made with Natural Earth.

Root-to-tip regression analysis confirmed the presence of a significant temporal signal in the sequences of both segments as follows: *R*^2^ = 0.41 for the L segment (*P* < 0.001) and 0.21 for the S segment (*P* < 0.001). These results are in line with previous analyses (11, 15). As a next step in the analysis, we performed a time-scaled phylogenetic reconstruction using BEAST. The majority of the sequences clustered with the main Nigerian lineages II and III (Fig. 1). None of the new sequences clustered with lineage I, the prototype Lassa virus strain from the town of Lassa. However, several sequences formed a new monophyletic cluster that is well supported (posterior values of 1 in both S and L tree) but shows an ambiguous relationship to lineages I, II, and III (different tree topologies and poor posterior values in S and L tree) (see Fig. S1 and S2 in the supplemental material). This cluster comprises the following two subclusters: (i) the Kako strains detected in *Hylomyscus pamfi* (12) and (ii) a novel full-length S and L segment sequence (LASV/H.sapiens-tc/NGA/2016/IRR_006 reported in this study) from Ekiti State as well as the short (0.3 kb) S segment sequences previously identified in patients from Edo State (Nig05-A08 and Nig09-045) (14) (clades a, b, and c in Fig. 1 and 2; see also Fig. S1 and S2).

Previous sequencing studies revealed that lineage III strains circulate in the north of Nigeria and lineage II strains are prevalent in the south (4, 14). While this geographical pattern still holds true, the new sequences facilitate a much higher spatial resolution regarding the circulation of sublineages within the main two lineages. We applied the following set of criteria to define sublineages: they (i) form a monophyletic clade with high posterior support in the S and/or L tree, (ii) originate from deeper nodes, and (iii) have been sampled in a confined geographic area (see Fig. S3, S4, S5, and S6 in the supplemental material for classification of the sublineages in the trees and Fig. S7 and S8 in the supplemental material for the respective sampling maps). These criteria have solely been established with the purpose to describe the currently available data; they are not meant as formal criteria for subclassification of lineages. Within lineage III, five sublineages (3a to 3e) were distinguishable (Fig. 1 and 2). Sublineage 3a was found in Plateau (*n* = 8), Kaduna (*n* = 4), Bauchi, Federal Capital Territory, and Benue (states are listed according to the number of sequences; *n* ≤ 2 is not indicated); 3b in Benue (*n* = 4) and Nasarawa; 3c in Nasarawa (*n* = 12), Federal Capital Territory (*n* = 3), and Plateau; 3d in Taraba; and 3e in Bauchi (*n* = 19) and Plateau (*n* = 3). Within lineage II, seven sublineages were distinguishable (2a to 2g) (Fig. 1 and 2). Sublineage 2a was found in Ebonyi (*n* = 3) and Abia; 2b in Imo (*n* = 3), Rivers (*n* = 3), Enugu, and Akwa Ibom; 2c in Ebonyi (*n* = 20) and Imo; 2d in Taraba (*n* = 5), Anambra, and Adamawa; 2e in Delta; 2f in Kogi (*n* = 3) and Anambra; and 2G in Edo (*n* = 46), Ondo (*n* = 41), Kogi (*n* = 3), Delta, Anambra, and Ekiti. Owing to the large number of sequences in sublineage 2g, it has even been possible to distinguish minor phylogenetic clusters in specific localities (see Fig. S5 and S6): 2g1 along the Niger River in Delta and Anambra; 2g2 around Uromi town in Edo; 2g3 around Owo and Ifon towns in Ondo; and 2g4 along the Niger River in Kogi. However, the matching of phylogenetic clusters with specific locations has to be interpreted with caution, as most viruses stem from patients and the precise location where the infection was acquired is not known. Indeed, the phylogenetic origin of a few sequences (mostly incomplete sequences marked with asterisks in Fig. S3, S4, S5, and S6) was implausible in view of the recorded origin of the patient. These sequences were excluded from the above mapping.

**FIG 3:**
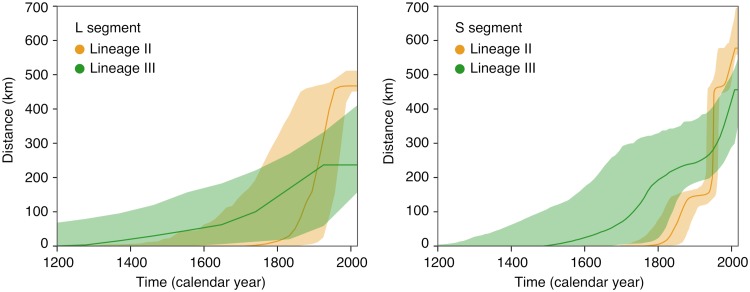
Evolution of maximal wavefront distance estimated for Lassa virus lineages II and III based on the continuous phylogeographic analysis of segments L and S. The plots display the temporal evolution of the maximal wavefront distance, i.e., the spatial distance between the furthest extent of the wavefront and the position of the most ancestral node.

**FIG 4:**
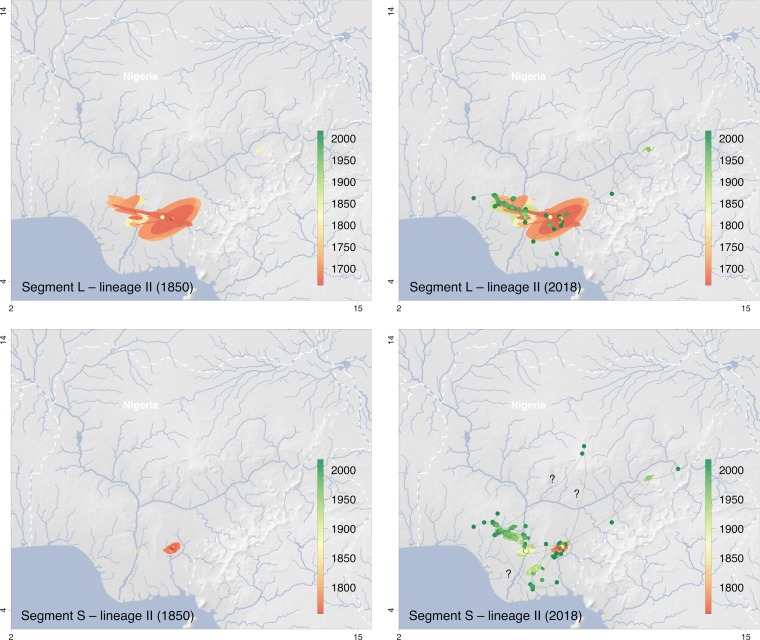
Snapshots of the spatiotemporal diffusion of Lassa virus lineage II estimated from continuous phylogeographic reconstructions based on segments L and S. The plots show temporal snapshots of the mapped maximum clade credibility (MCC) trees and 95% highest posterior density (HPD) regions based on 1,000 trees subsampled from the post burn-in posterior distribution of trees. Nodes of the MCC tree (dots) are colored according to a color scale ranging from red (the time to the most recent common ancestor) to green (most recent sampling time). The 95% HPD regions were computed for successive time layers and then superimposed using the same color scale reflecting time. Lines connecting the dots depict the direction of virus spread. Lines pointing to outlier sampling sites likely to be artifacts due to patient movement or sample mix-up are identified by a “?”; the respective sequences are marked with an asterisk in Fig. S5 in the supplemental material. The spatiotemporal diffusion is shown for S and L segment trees for the years 1850 and 2018. International borders and rivers are represented by white dashed lines and blue lines, respectively. Maps made with Natural Earth.

The geographic clustering of lineages and sublineages led us to attempt to reconstruct the spatial and temporal evolution of Lassa virus in Nigeria. The phylogeographic analysis estimated a weighted dispersal velocity of about 1 km per year and a spread into an area of about 50 km^2^ per year for both lineage II and III (Table 1). The dispersal of lineages II and III may have started approximately 300 and 800 years ago, respectively, though the estimations slightly differ for the S and L segment (Fig. 3). The snapshots of the spatial distribution of the virus over time as estimated using S and L segment-based phylogenies suggest an origin of lineage II in the southeastern part of the country around Ebonyi and a main vector of distribution toward the west across the Niger River, through Anambra, Kogi, Delta, and Edo into Ondo State (Fig. 4). Sublineages 2a and 2c still circulate in Ebonyi, while the movement toward the west has led to the evolution of sublineage 2f in Anambra and Kogi, 2e in Delta, and 2g in Edo and Ondo State. The frontline of virus movement appears to be in Ondo. However, a single sequence in 2g is from Ekiti, north of Ondo, and might be a first indication of virus movement into this state. Minor vectors of distribution are directed northeast toward Taraba and Adamawa (sublineage 2d) and south toward Imo, Rivers, and Akwa Ibom (sublineage 2b).

**TABLE 1:** Dispersal statistics for Lassa virus lineages estimated from continuous phylogeographic analyses^*a*^

Lineage	Mean branch dispersal velocity (km/yr)	Weighted dispersal velocity (km/yr)	Mean diffusion coefficient (km^2^/yr)	Weighted diffusion coefficient (km^2^/yr)
L segment				
Lineage II	4.08 [2.36–12.2]	1.33 [0.96–1.73]	67.7 [42.3–185]	49.4 [35.6–74.6]
Lineage III	2.21 [1.18–4.83]	0.92 [0.65–1.23]	64.6 [30.8–162]	38.6 [25.7–53.0]
S segment				
Lineage II	9.93 [4.66–39.2]	1.47 [1.23–1.73]	839.3 [291–4454]	85.4 [71.8–100]
Lineage III	3.19 [1.83–11.7]	1.04 [0.86–1.25]	101.2 [58.1–429]	43.4 [35.5–59.1]

a The table reports median value and 95% highest posterior density interval for each estimate. Mean branch velocity and diffusion coefficient are estimates over all tree branches. The weighted estimates involve a weighting by branch time.

**FIG 5:**
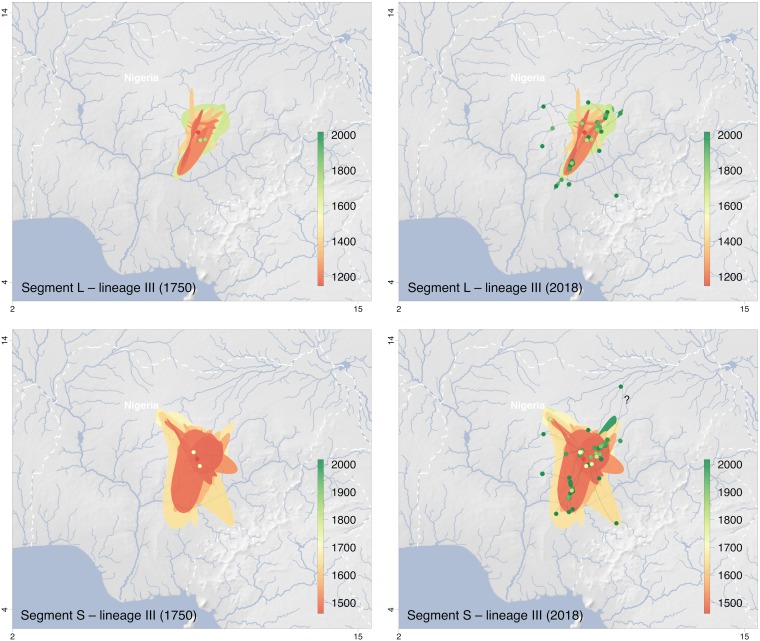
Snapshots of the spatiotemporal diffusion of Lassa virus lineage III estimated from continuous phylogeographic reconstructions based on segments L and S. The plots show temporal snapshots of the mapped maximum clade credibility (MCC) trees and 95% highest posterior density (HPD) regions based on 1,000 trees subsampled from the post burn-in posterior distribution of trees. Nodes of the MCC tree (dots) are colored according to a color scale ranging from red (the time to the most recent common ancestor) to green (most recent sampling time). The 95% HPD regions were computed for successive time layers and then superimposed using the same color scale reflecting time. Lines connecting the dots depict the direction of virus spread. Lines pointing to outlier sampling sites likely to be artifacts due to patient movement or sample mix-up are identified by a “?”; the respective sequences are marked with an asterisk in Fig. S3 in the supplemental material. The spatiotemporal diffusion is shown for S and L segment trees for the years 1750 and 2018. International borders and rivers are represented by white dashed lines and blue lines, respectively. Maps made with Natural Earth.

The origin of lineage III might be in northern Plateau State (Fig. 5). The model suggests a centrifugal spread of the virus into the neighboring states of Kaduna (sublineage 3a), Nasarawa/Federal Capital Territory (sublineage 3c), and Bauchi (sublineage 3e). While movement of these three sublineages apparently was limited to the territory north of Niger and Benue rivers, sublineage 3b has moved south and crossed the Benue River into Benue State (Fig. 2). This movement is well documented, as the rodents from which the sequences ONM-299, ONM-314, and ONM-700 were obtained had been trapped south of the river (12). Sublineage 3d also seemed to have crossed the Benue River and moved into Taraba State, although evidence is weak because only a single sequence for this cluster is available (Fig. 2).

## DISCUSSION

A geographical pattern in the distribution of Lassa virus was disclosed by the first comprehensive sequencing study on Lassa virus published in 2000 (4). It demonstrated a westward movement of the virus across West Africa and classified Lassa virus into lineages. Our study provides a geographic mapping of lineages and phylogenetic clusters in Nigeria at a higher resolution. In addition, we estimated the direction and time frame of virus dispersal in the country. Essentially, the data confirm the distribution of lineage III in the northern part of the country and lineage II in the southern part of Nigeria.

As the majority of human infections result from spillover of the virus from the natural reservoir rather than human-to-human transmission (11, 15), the geographical pattern of Lassa virus is primarily a consequence of the temporal and spatial evolution of the virus in the rodent population. The observation that sublineages and clusters are confined to separated geographical areas and are not dispersed and mixed over the Nigerian territory is consistent with the limited home range of *M. natalensis* (16, 17). On the other hand, *M. natalensis* populations also include mobile animals that may have driven virus dispersal on an evolutionary scale (16, 18). Landscape structures that facilitate or restrict rodent movement might have contributed to shaping the current distribution of lineages and sublineages (19). Specifically, the large Niger and Benue rivers may represent geographical barriers to the movement of rodents between the northern and southern parts of the country. However, previous ecology and our human studies indicate that infected rodents carrying lineage III viruses have crossed the Benue River from the north at least into Benue state (12). Lineage II, which appears to originate from the southeast has clearly crossed the Niger River and moved into the southwestern part of the country. Our model estimated an average speed of virus dispersal of about 1 km per year. The estimate for the molecular clock of 7.8 × 10^−4^ substitutions/(site × year) and time to most common recent ancestor of currently circulating strains in Nigeria of about 800 years are in agreement with previous studies (11, 14). However, estimates for temporal and spatial dispersal have to be interpreted with caution the deeper we look back in time. Our estimates are based on sampling over the past 50 years; however, recent phylogenetic studies that included sequences of ancient DNA and RNA viruses estimated substitution rates approximately 1 order of magnitude lower than rates inferred solely from modern sequences (20–22). Thus, it is conceivable that the velocity of virus dispersal is slower than that estimated here. It will be interesting to follow the dispersal of the virus at the frontline of lineage II, which currently seems to be in Ondo State, and experimentally challenge the estimates.

A main limitation and confounding factor of our study is that it is based on human cases attending hospitals rather than systematic sampling in the rodent population. Thus, there might be a sampling bias owing to the risk of rodent-to-human transmission in an area, the distance between villages with Lassa fever and the nearest secondary or tertiary hospital, the awareness of doctors in the local hospitals to suspect Lassa fever, the availability of diagnostic and treatment facilities, and the knowledge about the available options to test for Lassa fever at national reference laboratories. In addition, the mobility of patients may lead to a substantial distance between the site of infection and the site of presentation to a doctor, which often was the only available spatial information. Mixing up, mislabeling, or contamination of samples in the hospital or laboratory may also not be excluded and might have confounded the analysis. Phylogeographic inference remains conditioned on the sampling, and heterogeneous sampling density can result in more or less notable differences between the actual viral spread and the inferred dispersal history of lineages (23). The phylogeographic inference performed here aimed at reconstructing the ancestral history of viral lineages in a continuous space but is not necessarily a detailed picture of the dispersal history of the entire viral population, even if sampling would reflect the true density. Influencing factors such as landscape structures or adaptation to new hosts during evolution have not been considered in this model.

Besides the known lineages I, II, and III, we found a well-supported monophyletic cluster of sequences that does not fall within these lineages. This clade comprises the strains from a *H. pamfi* rodent trapped in Kako, southwestern Nigeria (12), as well as human sequences from the Ekiti (this study) and Edo states (14). Unfortunately, the sequences from Edo State are too short for an in-depth analysis of the relationship between the latter sequences. Formal analysis on whether the Kako and Ekiti sequences represent one or even two new Lassa virus lineages is pending. The relationship of Lassa virus in *M. erythroleucus* and *H. pamfi* with lineage III and the new cluster, respectively, raises the question of whether specific lineages or sublineages of Lassa virus are associated with specific host species. Although *M. natalensis* is considered the principal host of Lassa virus, this association has been mainly confirmed for lineages II and IV and may not hold true for other lineages or sublineages (9–11, 13). Besides the rare outlier strains from patients from Ekiti (*n* = 1) and Edo (*n* = 2), lineage I has been observed only once during a nosocomial outbreak in 1969 in the town of Lassa in the north of the country and never again. It is conceivable that the natural hosts of these rare strains or lineages are not commensal rodents living close to humans, such as *M. natalensis*, but wild rodents, which usually do not have contact to humans, such as *H. pamfi*. If host switching were involved in the evolution of Lassa virus lineages, this would increase the complexity and timelines of the spatiotemporal evolution of the virus.

This project not only aimed at generating Lassa virus sequences from across Nigeria but also at isolating and conserving the respective viruses for future research. This work has been performed by a Nigerian fellow (D. U. Ehichioya) in a biosafety level 4 facility in Hamburg. Besides their use in studies on virus evolution, the sequences and isolates are relevant to public health research. For example, they have been important for the development and evaluation of the RealStar Lassa virus reverse transcriptase PCR (RT-PCR) kits (altona Diagnostics, Hamburg, Germany), which are now a diagnostic reference assay in Nigeria. In addition, they served as background information for recent sequencing studies in Nigeria, demonstrating that the high incidences of Lassa fever cases in 2018 and 2019 are not associated with the circulation of newly emerging strains or lineages or increased human-to-human transmission (15) (http://virological.org/t/2019-lassa-virus-sequencing-in-nigeria-final-field-report-75-samples/291).

## MATERIALS AND METHODS

### Samples.

Leftover Lassa virus RT-PCR-positive samples from Lassa fever diagnostics at ISTH were employed in this study. These were selected based on the geographical origin of the sample and high genetic diversity as observed from the preliminary sequence analysis of short PCR fragments. The ISTH and National Health Research and Ethics Committees approved the use and transfer to Hamburg of these samples and corresponding patient data (approval numbers ISTH/HREC/20171208/45 and NHREC/01/01/2007-5/02/2018, respectively).

### Sequencing.

Full-length or partial Lassa virus sequences were generated from cell culture isolates or directly from patient samples using NGS and Sanger sequencing. Virus was isolated and propagated on Vero cells in a biosafety level 4 laboratory. Supernatant was harvested on day 3 after passage and used for Lassa virus sequencing. If Lassa virus could not be cultured, sequencing was attempted directly from clinical specimens. Supernatant or clinical sample was centrifuged and passed through a 0.45-μm filter (Millipore) to remove cellular debris and bacterial-cell-sized particles. Filtrates were treated with a cocktail of nucleases, namely, Turbo DNase (Ambion), Benzonase (Novagen), Baseline-Zero DNase (Epicentre), and RNase A (ThermoFisher Scientific), to eliminate contamination from non-particle-protected nucleic acids, bacteria, and eukaryotic cells (24, 25). RNA was extracted from filtrates by using the QIAamp viral RNA minikit (Qiagen) or the MagMax total RNA isolation kit (MagMax; Ambion) according to the manufacturer’s instruction with few modifications. Extracted RNA was treated with RNase inhibitor (Invitrogen) for protection against degradation. Viral cDNA was synthesized from 6 μl of viral RNA by incubating with random octamer primer (26), SuperScript III reverse transcriptase (Invitrogen), deoxynucleoside triphosphates (dNTPs), dithiothreitol (DTT), and first-strand buffer. Complementary strand synthesis was performed using Klenow fragment polymerase (New England Biolabs) with an additional 20 pmol of the same primers. The products were purified using Agencourt AMPure XP beads (Beckman Coulter), and purified double-stranded DNA (dsDNA) was subsequently PCR amplified using primers consisting of the fixed portions of the random primer. PCR products were subjected to combined purification and size selection, again with the Agencourt AMPure XP beads (Beckman Coulter), and quantified by fluorimetry with the Qubit dsDNA high-sensitivity assay on the Qubit 3.0 instrument (Life Technologies). The Nextera XT DNA sample preparation kit (Illumina) and 1 ng dsDNA were used to generate sequencing libraries according to the manufacturer’s guidelines. Briefly, the dsDNA was fragmented, tagged with adapter and dual-indexed, and amplified in a limited 15-cycle PCR. Libraries were then purified and analyzed on a high-sensitivity DNA chip on the Bioanalyzer (Agilent Technologies) for the sequence length distribution. All libraries were then pooled to an equal concentration. Shortly before sequence run, the library pool was denatured and further diluted to the desired concentration, usually 10 pM. A control library (1% PhiX library; Illumina) was added to the pool and loaded onto the flow cell of the 600-cycle MiSeq reagent kit v3 (Illumina) and sequenced on the Illumina MiSeq platform. Quality check on Illumina raw sequence data was performed with FastQC (available at http://www.bioinformatics.babraham.ac.uk/projects/fastqc/). Reads were filtered and trimmed for adapters and contaminants using the BBDuk tools (available at https://jgi.doe.gov/data-and-tools/bbtools/) and then compared against the NCBI nonredundant database with blastx. Genome finishing, sequence assembly, and analysis were performed using Geneious v9.1.5. (Auckland, New Zealand). If Lassa virus isolation or Illumina sequencing failed, Lassa virus genome fragments were amplified from clinical samples of patients by RT-PCR using primers targeting the S segment (14, 27) and sequenced with the Sanger method.

### Metadata collection.

Metadata for phylogeographic inference included the date of sampling (year, month, day) and the place of residence of the patient (Nigerian state, town/village), which was taken as the sampling site of the virus. Minimum information for inclusion of a strain in the analysis was year of sampling and the patient’s state of residence. If the hometown or village of a patient was not known, the hospital, which was attended by the patient or where the diagnostic specimen had been taken, was used as a proxy. Metadata for the Lassa virus strains sequenced in this study were retrieved from the laboratory request forms that accompanied the specimens. In addition, information was gathered through interaction with the submitter of the specimen. To enlarge the sample size, we also included Lassa virus sequences in the analysis, which we sequenced in 2018 from clinical specimens processed in the diagnostic laboratory at ISTH (NCBI BioProject accession number PRJNA482058) (15). To facilitate inclusion of these samples, we retrieved detailed information on the sampling sites from ISTH files. Metadata for other previously published sequences were obtained from published sources (see References in Table S1 in the supplemental material). For nosocomial transmission chains, a single representative strain was included, and date and place of the index case was used. Geographical coordinates, i.e., latitude and longitude for towns and villages (*n* = 201) were gathered from various sources (https://geographic.org/; https://www.google.com/maps; https://www.latlong.net/; http://www.maplandia.com/; https://www.wikipedia.org/). If only the local governmental area of a patient’s residence was known (*n* = 11), the centroid point of the polygon representing that area in the GADM database (https://gadm.org/) was taken as a sampling point. If only the patient’s state of residence was known (*n* = 7), the median coordinates of all of the other strains sampled within the same state were used. For strains from rodents, coordinates of the reported trapping sites were taken as the sampling sites. The final data set for phylogeographic inference included sequences (*n* = 216 for S segment and *n* = 157 for L segment) and metadata for 219 unique Lassa virus strains (*n* = 77 from this study [NCBI BioProject accession number PRJNA482054]; *n* = 101 from our 2018 sequencing study [NCBI BioProject accession number PRJNA482058]; and *n* = 41 from other sources [GenBank, accessed September 2018]). Metadata, accession numbers, and references for all Lassa virus strains/isolates used in this study are listed in Table S1.

### Temporal signal analysis.

For each segment, we evaluated the phylogenetic temporal signal using regressions of root-to-tip genetic distances against sequence sampling times. The analyses were based on maximum likelihood trees inferred with the program FastTree 2 (28), and the determination coefficients (*R*^2^) of the linear regression were estimated with the program TempEst (29). The *P* values were calculated using the approach of Murray et al. (30) and based on 1,000 random permutations of the sequence sampling dates (31).

### Continuous phylogeographic analysis.

Continuous phylogeographic inferences were performed using BEAST 1.10.4 (32) and the BEAGLE library (33) to improve computational performance. As reassortment is known to occur between the two Lassa virus genome segments (11, 15), we performed separate analyses for both the L and S segment data, resulting in two separate analyses of the same lineage dispersal history. The substitution processes were modelled according to a GTR + Γ parametrization (34). A flexible skygrid coalescent model was specified as tree topology prior (35), and relaxed clock models with rates drawn from an underlying lognormal distribution (36) were fit for both segments. For computational reasons, preliminary BEAST analysis to investigate the evolutionary relationships among lineages was based on a simple constant population size model. We used the relaxed random walk diffusion model (RRW) (37) to generate a posterior distribution of trees whose internal nodes are associated with inferred geographic coordinates. Markov chain Monte Carlo (MCMC) chains were run for more than 850 and 240 million iterations for segments L and S, respectively. The chains were sampled every 100,000 generations, and the first 200 (L segment) and 500 (S segment) sampled trees were removed as burn-in. Convergence and mixing properties were inspected using Tracer 1.7 (38) to ensure that estimated sampling size (ESS) values associated with estimated parameters were all >200. For both segments, maximum clade credibility (MCC) trees were summarized using TreeAnnotator 1.10.4 (32) from 1,000 trees regularly sampled from each posterior distribution. We used the R package “seraphim” (39, 40) to extract the spatiotemporal information embedded within the same 1,000 posterior trees. We further used this package to estimate the following dispersal statistics based on these trees: the mean branch dispersal velocity, the weighted branch dispersal velocity, the mean diffusion coefficient (41), and the weighted diffusion coefficient (42) (see the package manual for the formulas and further details about these statistics). These dispersal statistics were estimated for each segment and while considering lineages II and III separately (4). Finally, the package “seraphim” was also used to estimate the evolution of the maximal wavefront distance (39, 40).

### Data availability.

Lassa virus S and L segment sequences (*n* = 141) generated in this study have been deposited with NCBI under BioProject accession number PRJNA482054.

## Supplementary Material

Supplemental file 1

Supplemental file 2
