# Supplementary material for: Phylogeography of Lassa Virus in Nigeria
Source: J Virol. 2019 Oct 15;93(21):e00929-19. doi: 10.1128/JVI.00929-19 (PMC6803284; doi:10.1128/JVI.00929-19)
Supplement: Supplemental file 1 [file JVI.00929-19-s0001.pdf]

Supplemental Figures

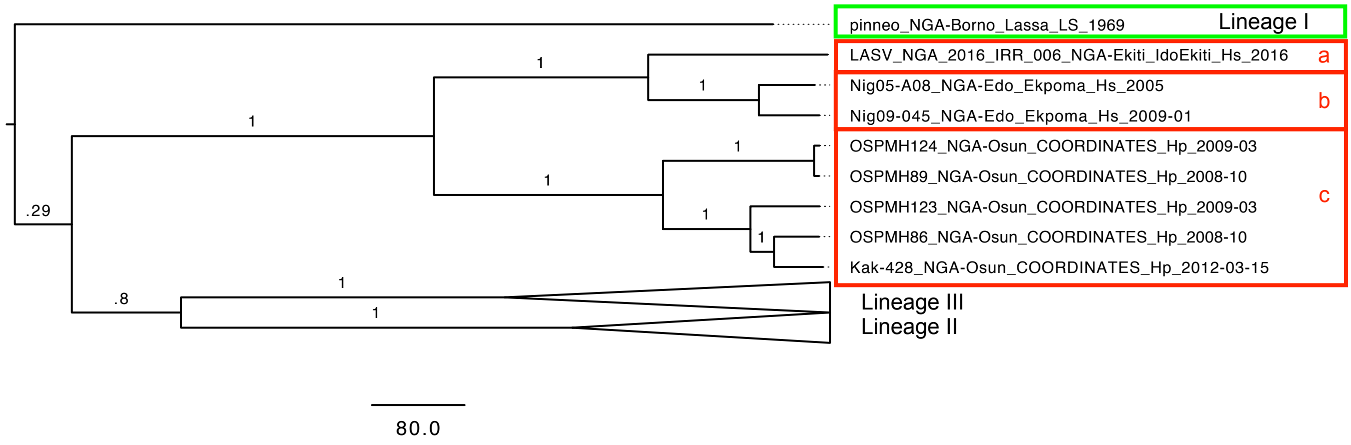

Figure S1. Time-scaled maximum clade credibility tree of outlier lineage I and related cluster (a, b, c) based on S segment. Lineages II and III are collapsed. Posterior support values are indicated on the branches. Values <0.8 are not shown on terminal branches. The strain designation includes country code (NGA for Nigeria), State, Town, species (Hs, *Homo sapiens*; Hp, *Hylomyscus pamfi*), and date of sampling. Further information on the sequences including geographical coordinates is provided in the Supplemental Data File. The scale bar indicates time in years.

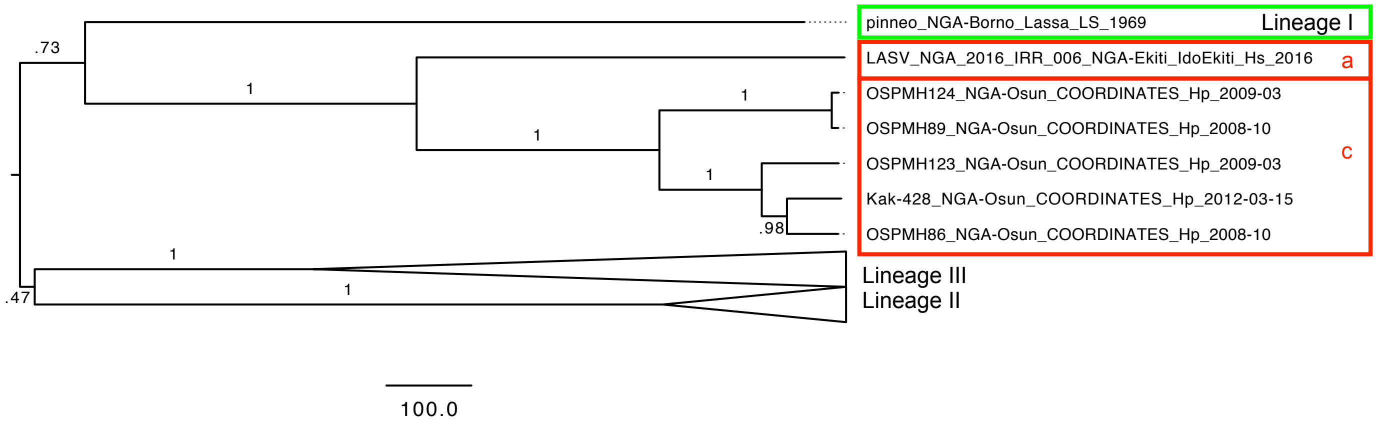

Figure S2. Time-scaled maximum clade credibility tree of outlier lineage I and related cluster (a, c) based on L segment. Lineages II and III are collapsed. Posterior support values are indicated on the branches. Values <0.8 are not shown on terminal branches. The strain designation includes country code (NGA for Nigeria), State, Town, species (Hs, *Homo sapiens*; Hp, *Hylomyscus pamfi*), and date of sampling. Further information on the sequences including geographical coordinates is provided in the Supplemental Data File. The scale bar indicates time in years.

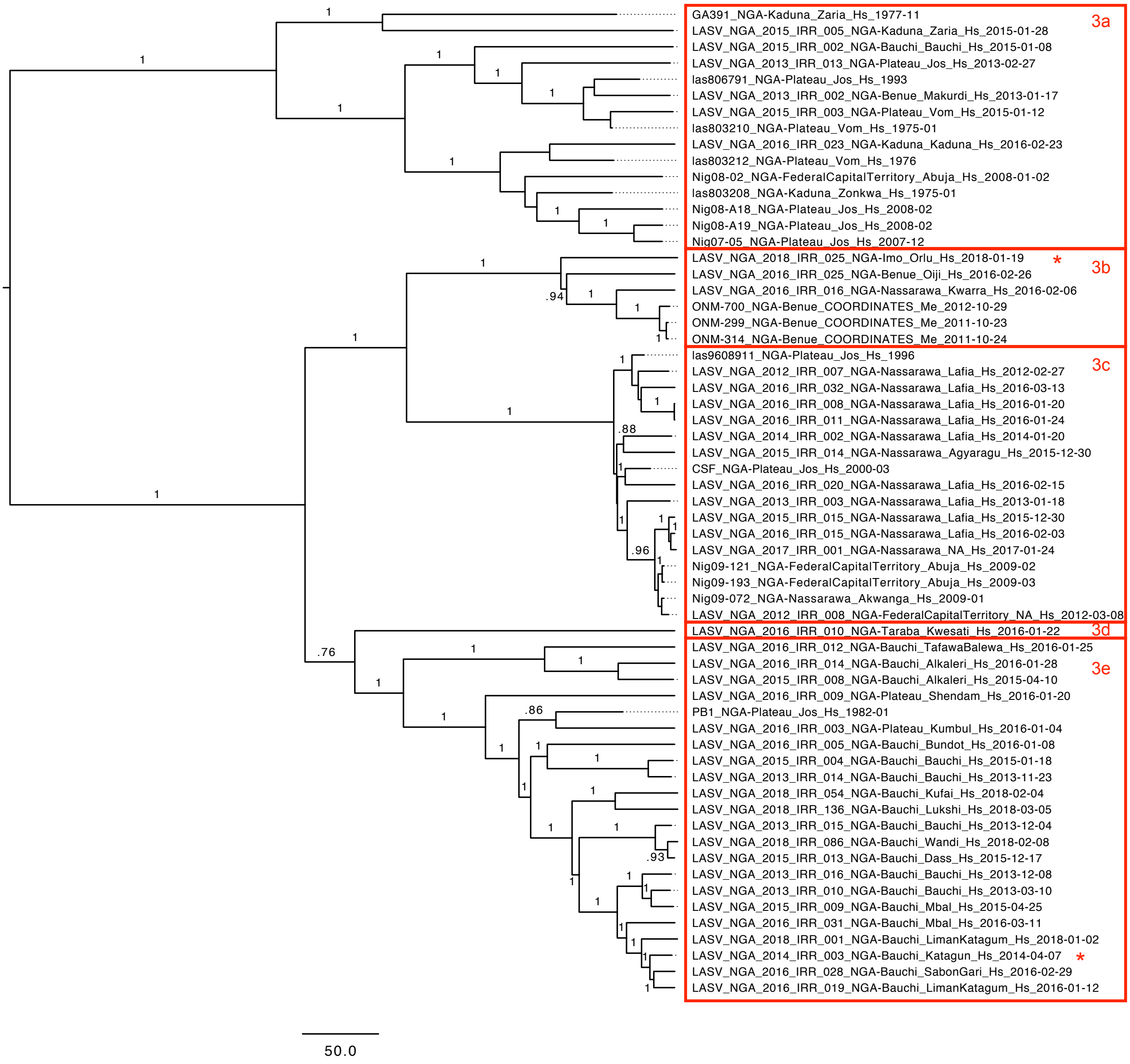

Figure S3. Time-scaled maximum clade credibility tree of lineage III based on S segment. Posterior support values are indicated on the branches. Values <0.8 are not shown on terminal branches. The strain designation includes country code (NGA for Nigeria), State, Town, species (Hs, *Homo sapiens*; Me, *Mastomys erythroleucus*), and date of sampling. Further information on the sequences including geographical coordinates is provided in the Supplemental Data File. Sub-lineages 3a–3e are depicted by boxes. Sequences with outlier sampling locations, i.e. locations that are far from the geographic core area of the respective sub-lineage, are marked with an asterisk. The scale bar indicates time in years.

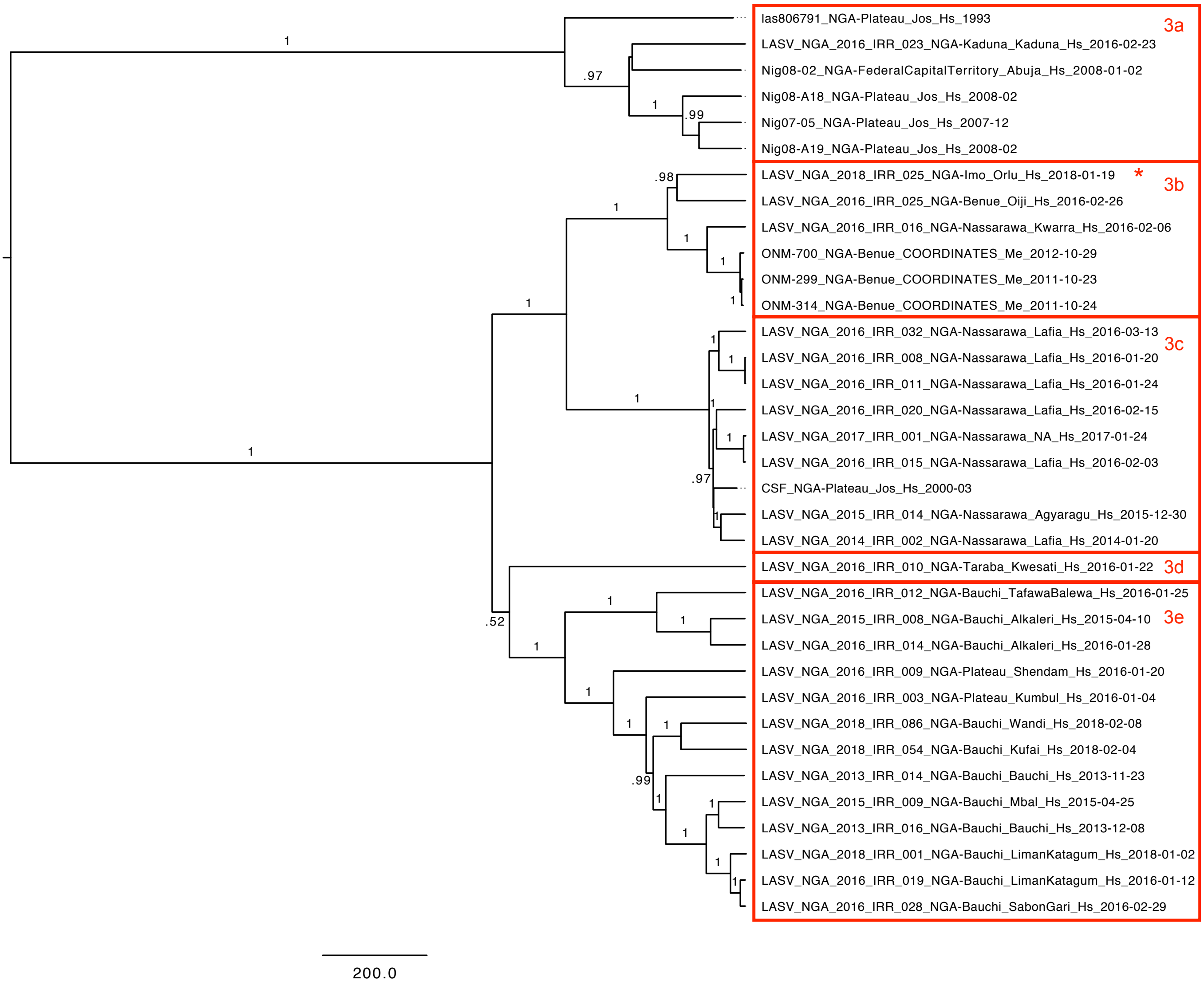

Figure S4. Time-scaled maximum clade credibility tree of lineage III based on L segment. Posterior support values are indicated on the branches. Values <0.8 are not shown on terminal branches. The strain designation includes country code (NGA for Nigeria), State, Town, species (Hs, *Homo sapiens*; Me, *Mastomys erythroleucus*), and date of sampling. Further information on the sequences including geographical coordinates is provided in the Supplemental Data File. Sub-lineages 3a–3e are depicted by boxes. Sequences with outlier sampling locations, i.e. locations that are far from the geographic core area of the respective sub-lineage, are marked with an asterisk. The scale bar indicates time in years.

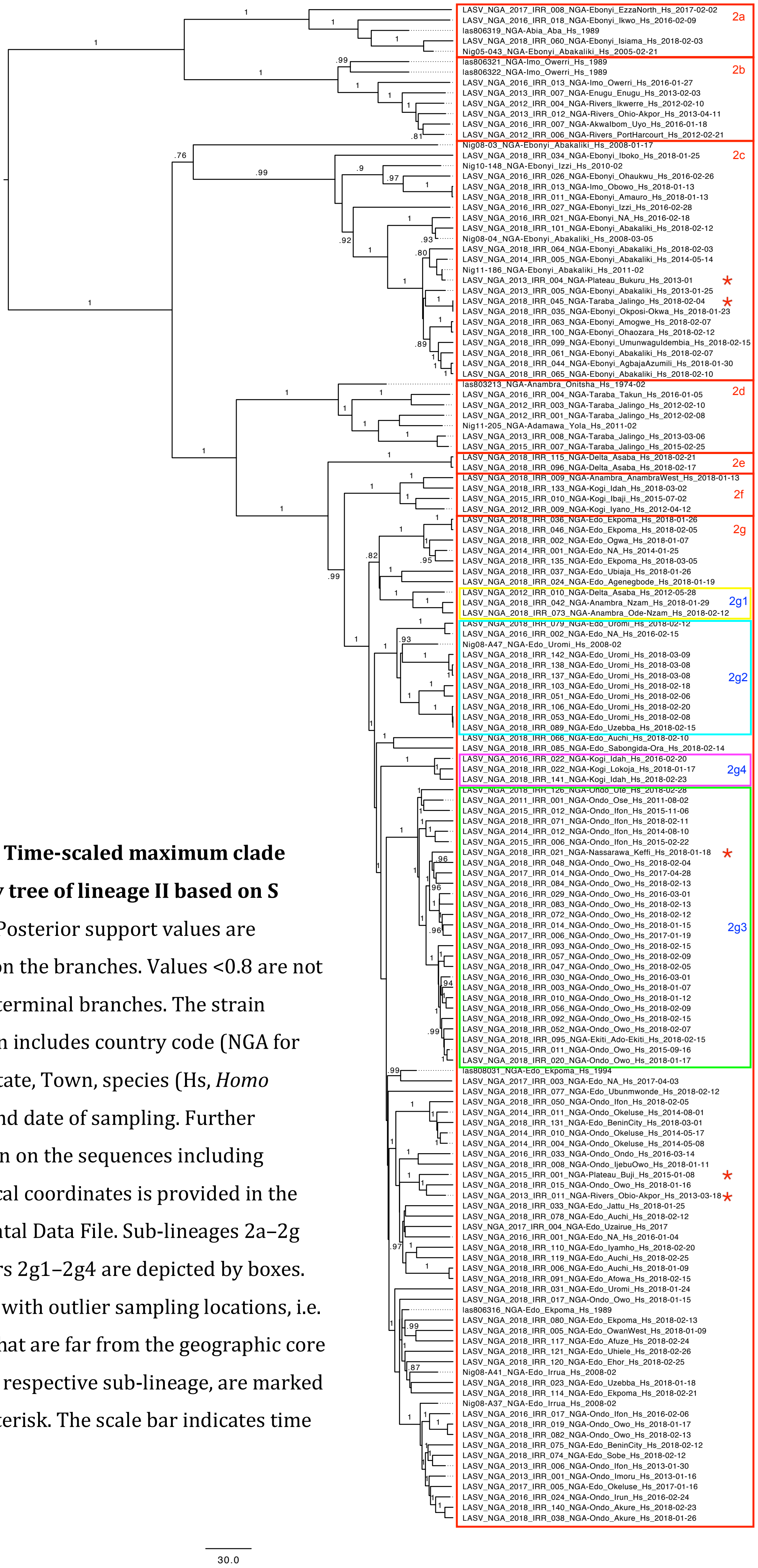

Figure S5. Time-scaled maximum clade credibility tree of lineage II based on S segment. Posterior support values are indicated on the branches. Values <0.8 are not shown on terminal branches. The strain designation includes country code (NGA for Nigeria), State, Town, species (Hs, *Homo sapiens*), and date of sampling. Further information on the sequences including geographical coordinates is provided in the Supplemental Data File. Sub-lineages 2a–2g and clusters 2g1–2g4 are depicted by boxes. Sequences with outlier sampling locations, i.e. locations that are far from the geographic core area of the respective sub-lineage, are marked with an asterisk. The scale bar indicates time in years.

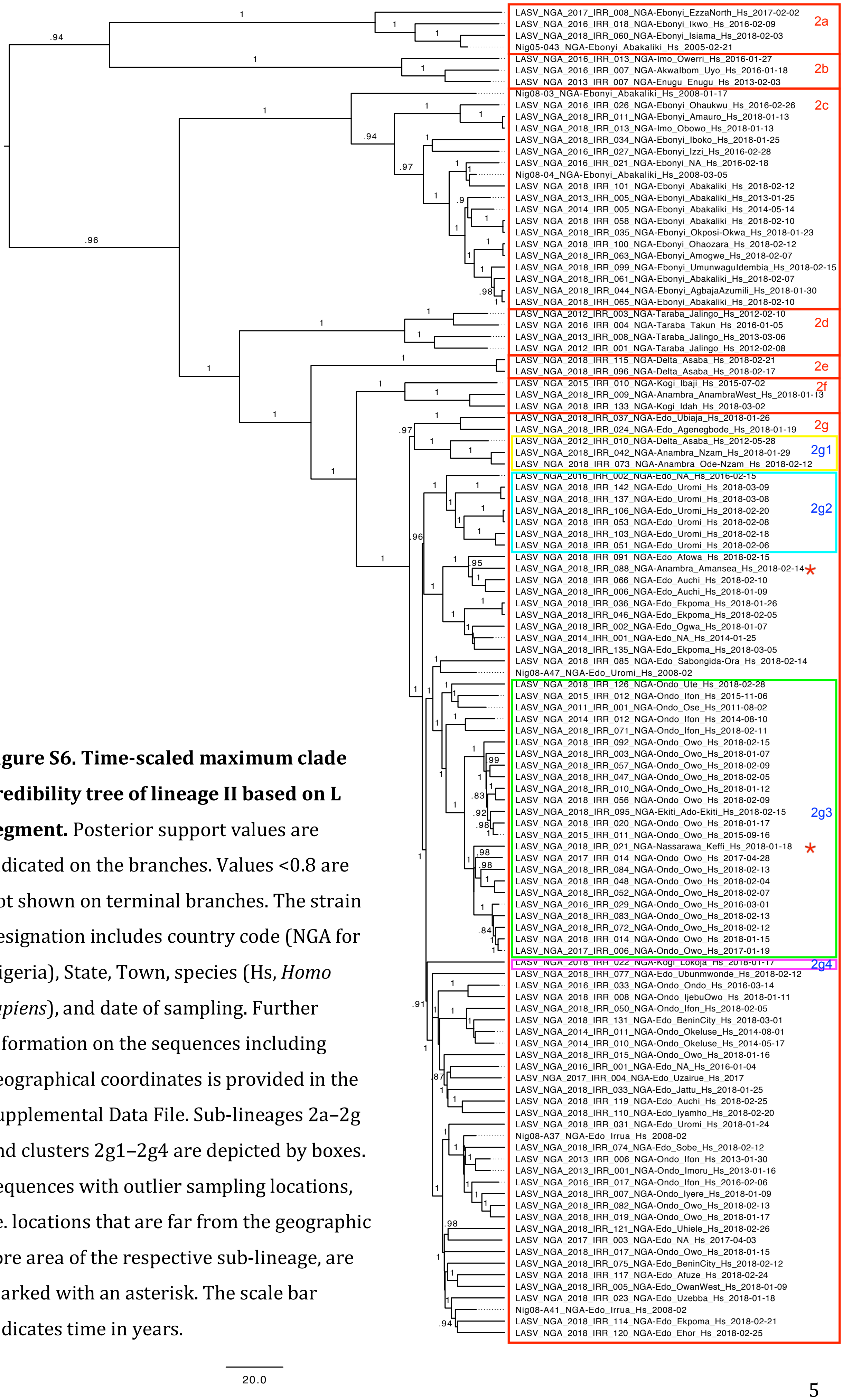

Figure S6. Time-scaled maximum clade credibility tree of lineage II based on L segment. Posterior support values are indicated on the branches. Values <0.8 are not shown on terminal branches. The strain designation includes country code (NGA for Nigeria), State, Town, species (Hs, *Homo sapiens*), and date of sampling. Further information on the sequences including geographical coordinates is provided in the Supplemental Data File. Sub-lineages 2a–2g and clusters 2g1–2g4 are depicted by boxes. Sequences with outlier sampling locations, i.e. locations that are far from the geographic core area of the respective sub-lineage, are marked with an asterisk. The scale bar indicates time in years.

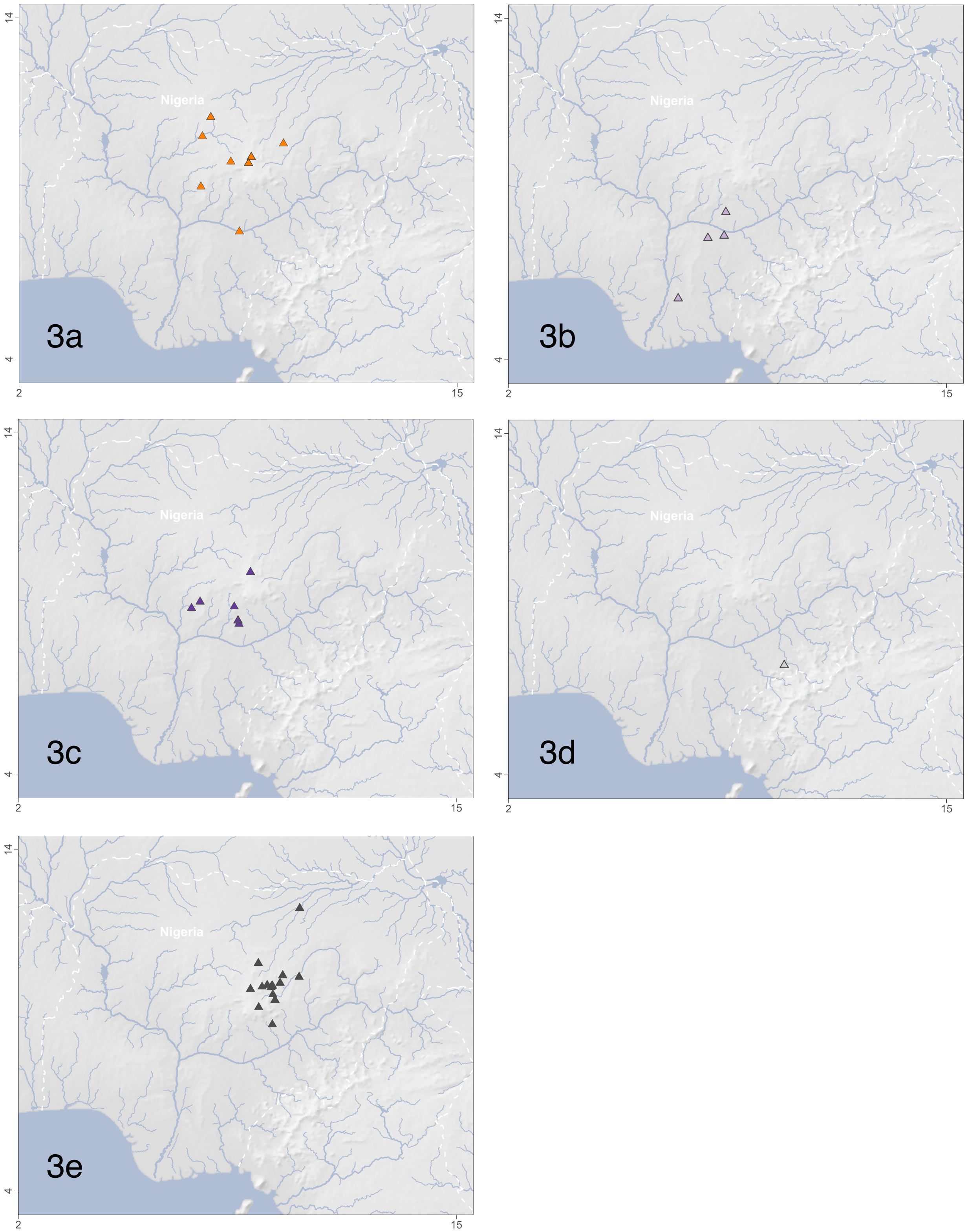

Figure S7. Sampling maps for sub-lineages within lineage III. Sub-lineages 3a–3e are defined in Figures S3 and S4. Each triangle represents a single strain or several strains with the same coordinates (see Supplemental Data File).

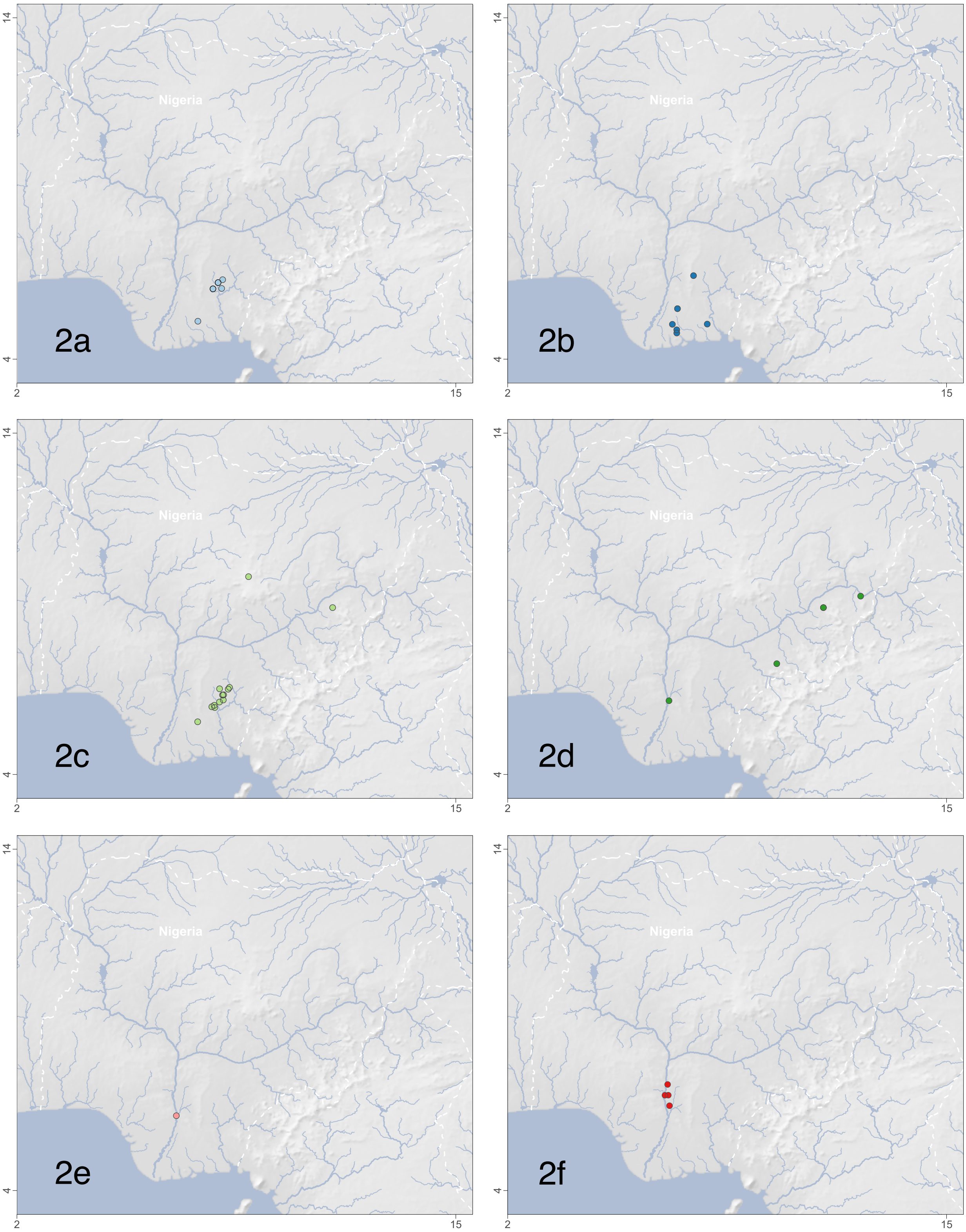

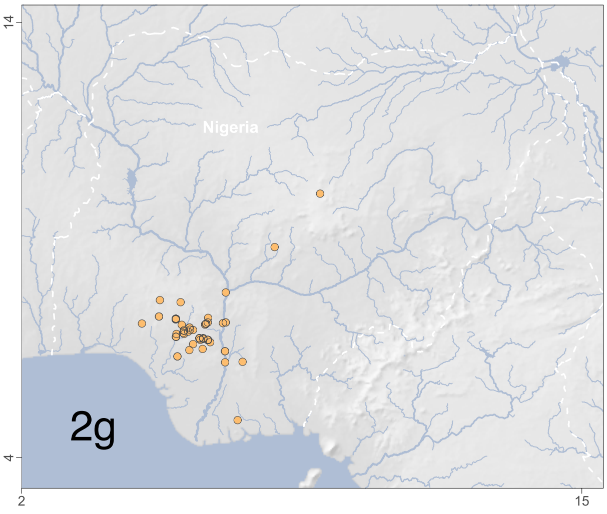

Figure S8. Sampling maps for sub-lineages within lineage II. Sub-lineages 2a–2g are defined in Figures S5 and S6. Each dot represents a single strain or several strains with the same coordinates (see Supplemental Data File).
